# Tracing the Vedic Saraswati River in the Great Rann of Kachchh

**DOI:** 10.1038/s41598-017-05745-8

**Published:** 2017-07-14

**Authors:** Nitesh Khonde, Sunil Kumar Singh, D. M. Maurya, Vinai K. Rai, L. S. Chamyal, Liviu Giosan

**Affiliations:** 1 Department of Geology, The M. S. University of Baroda, Vadodara, 390002 India; 2 Physical Research Laboratory, Navrangpura, Ahmedabad 380009 India; 3 Geology and Geophysics, Woods Hole Oceanographic Institution, Woods Hole, USA; 4 Birbal Sahni Institute of Palaeosciences, Lucknow, 266007 India

## Abstract

The lost Saraswati River mentioned in the ancient Indian tradition is postulated to have flown independently of the Indus River into the Arabian Sea, perhaps along courses of now defunct rivers such as Ghaggar, Hakra and Nara. The persistence of such a river during the Harappan Bronze Age and the Iron Age Vedic period is strongly debated. We drilled in the Great Rann of Kachchh (Kutch), an infilled gulf of the Arabian Sea, which must have received input from the Saraswati, if active. Nd and Sr isotopic measurements suggest that a distinct source may have been present before 10 ka. Later in Holocene, under a drying climate, sediments from the Thar Desert probably choked the signature of an independent Saraswati-like river. Alternatively, without excluding a Saraswati-like secondary source, the Indus and the Thar were the dominant sources throughout the post-glacial history of the GRK. Indus-derived sediment accelerated the infilling of GRK after ~6 ka when the Indus delta started to grow. Until its complete infilling few centuries ago, freshwater input from the Indus, and perhaps from the Ghaggar-Hakra-Nara, probably sustained a productive marine environment as well as navigability toward old coastal Harappan and historic towns in the region.

## Introduction

**Figure 1:**
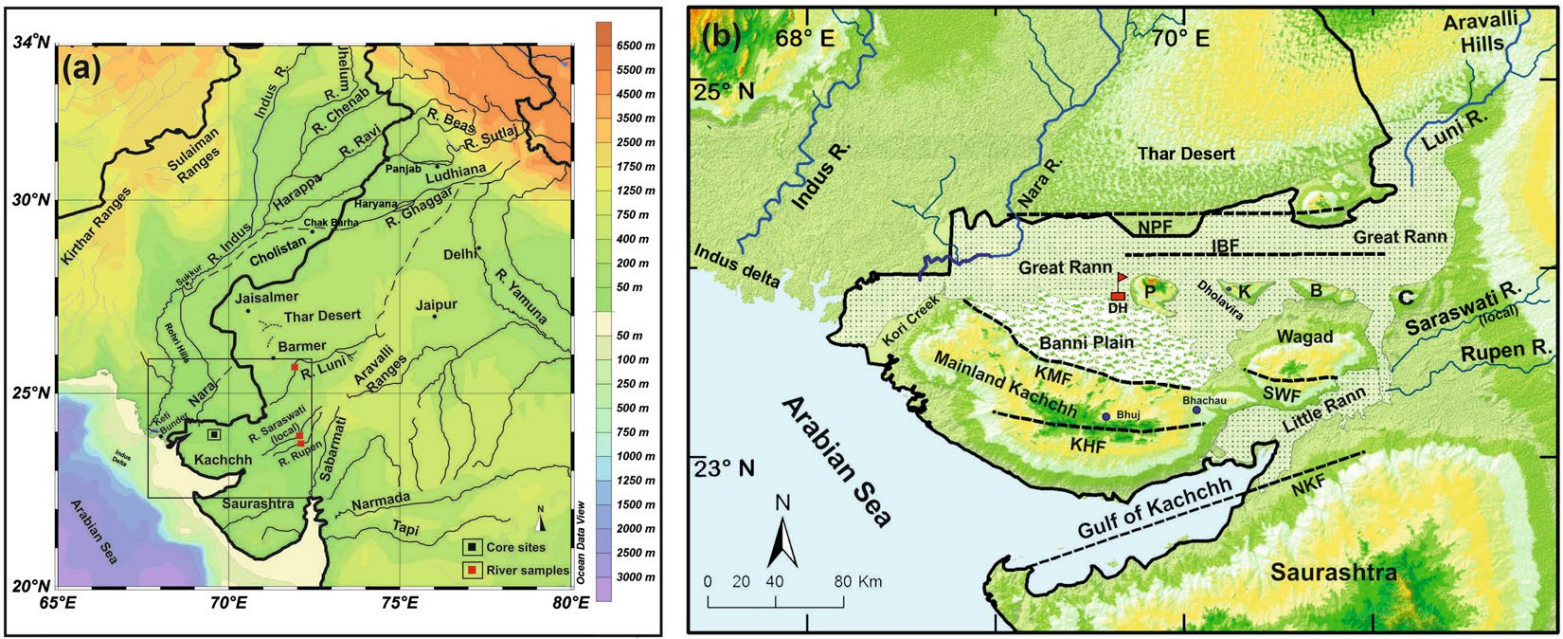
(**a**) Regional drainage pattern for the western continental margin of the Indian plate. Dotted lines are the paleochannels of the Vedic Saraswati River after Ghose *et al*.^11^ and Kar and Ghose^48^. The box represents the area shown in b. Location of the Dhordo core site and river sediment samples analyzed are also shown. (**b**) Geomorphic setting of the Great Rann of Kachchh (GRK) basin with surrounding hinterland and core locations. NPF- Nagar Parkar Fault, IBF- Island Belt Fault, KMF- Kachchh mainland Fault, KHF- Katrol hill Fault, NKF- North Kathiawar Fault, SWF- South Wagad Fault, P- Pachham Island, K-Khadir Island, B- Bela Island and C- Chorar Island. Core location: DH- Dhordo core raised from central GRK basin. Maps were prepared using a licensed copy of Ocean Data^49^ View (https://odv.awi.de/).

The Great Rann of Kachchh (GRK) is a landlocked and largely infilled shallow marine basin connected to the Arabian Sea, neighboring the Indus delta to the east. Thar Desert and Aravalli Hills border GRK to the north and northeast respectively (Fig. 1a and b). At present GRK is a monotonous, salt-encrusted, vast mudflat, largely dried up during early summer (i.e., March–July) and inundated during the summer monsoon and winter season (i.e., July to February). Strong summer monsoon winds push seawater from the Arabian Sea into the GRK; usually the water does not evacuate or evaporate until the next summer^1, 2^. Owing to the harsh conditions, lack of accessibility and limited sediment exposure, only a few geomorphological and geoarchaeological studies are available for the region^1–6^.

Several sites of the Bronze Age Harappan civilization flourished in and around GRK, including the large town of Dholavira on Khadir Island (Fig. 1b). As the Harappan coastal economy was probably dependent on maritime trade, the history of these towns was controlled by access to the Arabian Sea, which in turn was dependent on local sea level and fluvial infilling of the GRK^7, 8^. Previous studies postulated that a now extinct Vedic Saraswati River sourced in the Himalaya^9^ or Sub-Himalaya^8, 10^ reached down into the Arabian Sea as an independent river, parallel to the Indus^11, 12^. Such a river must have discharged into the paleo-gulf of GRK to reach the Arabian Sea. However, in spite of its geological and archaeological significance, GRK remains one of the least investigated regions of the Harappan domain^13^.

One of the keys to understand the geological and geomorphological evolution of the GRK is to fingerprint and resolve its potential sediment sources. Such sources may include the Indus River, the postulated Saraswati, the Arabian Sea shelf, the mainland Kachchh, Thar Desert and the Aravalli Ranges. In the present study we reconstruct sediment sources for the past ~17 ka^6^ using radiogenic tracers (i.e., Nd and Sr isotopes) in sediment core recovered from the GRK. The main goal of our study is to assess whether a Himalayan/Sub-Himalayan river reached the GRK independently of the Indus and for how long such a river was active, if at all.

Neodymium and strontium isotopes are some of the most robust provenance proxies. Nd is undergoing negligible alteration during erosion, sediment transport and deposition^14–17^ whereas Sr has been shown to be a good indicator of provenance in our study area^18, 19^. In our regional context, such studies have proven useful to explore erosion patterns, transport pathways, and provenance shifts for the Ganga–Brahmaputra^17, 20, 21^ as well as the Indus^19, 22^ fluvial systems. In addition, terrains neighboring the GRK such as the Thar Desert^23–25^, outcropping volcanics^26^ and Mesozoic rocks on Kachchh mainland^27^ have also been investigated for their Nd-Sr isotopic compositions.

## Results

The sediment core was recovered from the GRK (Fig. 1) near Dhordo village (23°49′37.9“N; 69°39′09“E) from the central Kachchh basin. Based on our previously published radiocarbon dates^28^, the Dhordo core recovered sediments as old as 17.7 ka down to ~60 m from the present day Rann surface. The subsurface GRK sediments studied in our core are consistently fine-grained in nature (i.e., silty-clay to clayey silts with negligible sand content; SI Fig. 1). Fine-grained sediments are typical for the GRK mudflats and remarkably consistent spatially and temporally in the entire basin^1, 3, 6, 29^. Our core at Dhordo is located far off from the elevated regions of mainland Kachchh, outside significant local sediment input, thus representing GRK basin wide changes. We assume that sedimentation is still active or non-erosional at Dhordo as the site is inundated during the summer monsoon.

The Dhordo core shows ^87^Sr/^86^Sr ratios range from 0.725 to 0.732 whereas ε_Nd_ varies from −14.34 to −12.63. (SI Table 1). To characterize potential end members, we also analyzed modern sediments from three local rivers, namely the Luni, Rupen as well as a local stream called Saraswati (no connection with the Vedic counterpart). The sample from the Luni River, which flows through the Thar yields ^87^Sr/^86^Sr and ε_Nd_ values of as 0.73 and −13.97. The local Saraswati stream and the Rupen River draining the Aravalli Hills yielded ^87^Sr/^86^Sr and ε_Nd_ values of 0.735, 0.731 and −15.22, −14.86 respectively (SI Table 1). For other sediment sources such as the fluvial or eolian sediments along the proposed Vedic Saraswati, the Indus courses and shelf as well as the Thar Desert we discuss published data below.

## Discussion

Presence of foraminifera throughout the core section indicates marine sedimentation throughout^6, 30^. To allow for fine-grained marine sedimentation at ~18 ka^30^, when the eustatic sea level in the Arabian Sea was below 100 m relative to present level^31, 32^, the Dhordo site must have been uplifted significantly since then. GRK is largely compressional and uplift of ca. 5 m is recorded for a marine sedimentary sequence on Khadir Island in the last 500 years^5^, so uplift at Dhordo is not surprising.

### Sediment Provenance

**Figure 2:**
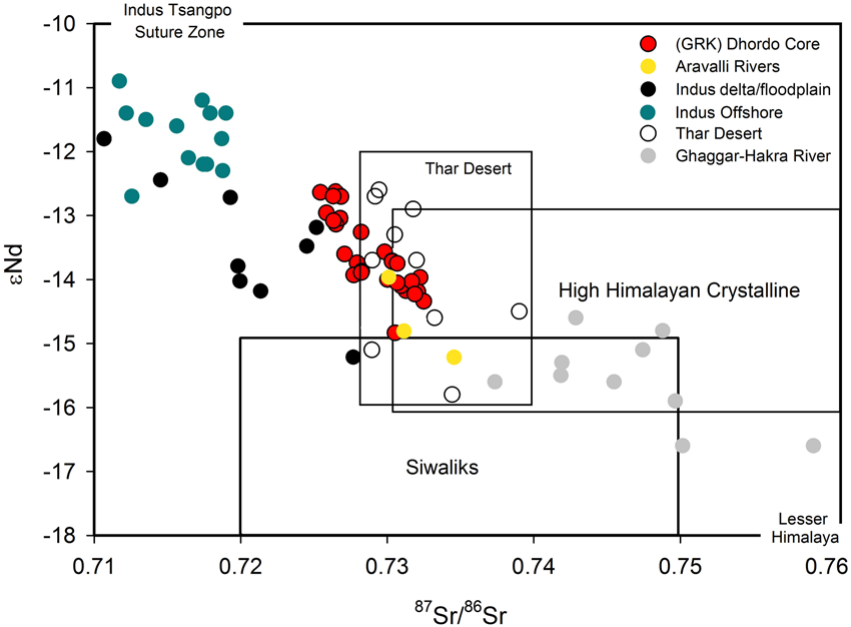
Scatter plot of ^87^Sr/^86^Sr and ε_Nd_ isotope compositions of our GRK sediments, Indus delta/floodplain (Clift *et al*.^18, 22^), Aravalli rivers (present study), Thar Desert (Tripathi *et al*.^25^), Ghaggar-Hakra fluvial system (East *et al*.^23^; Singh *et al*.^33^; Alizai *et al*.^50^ and the Indus shelf northwest of the Indus Canyon (Limmer *et al*.^19^) along with various potential end-members such as High Himalayan Crystalline, Lesser Himalaya and Siwaliks (Singh *et al*.^17^; Tripathi *et al*.^24, 25^ and references therein). Graph was prepared using a licensed copy of Sigma Plot v.10.

**Figure 3:**
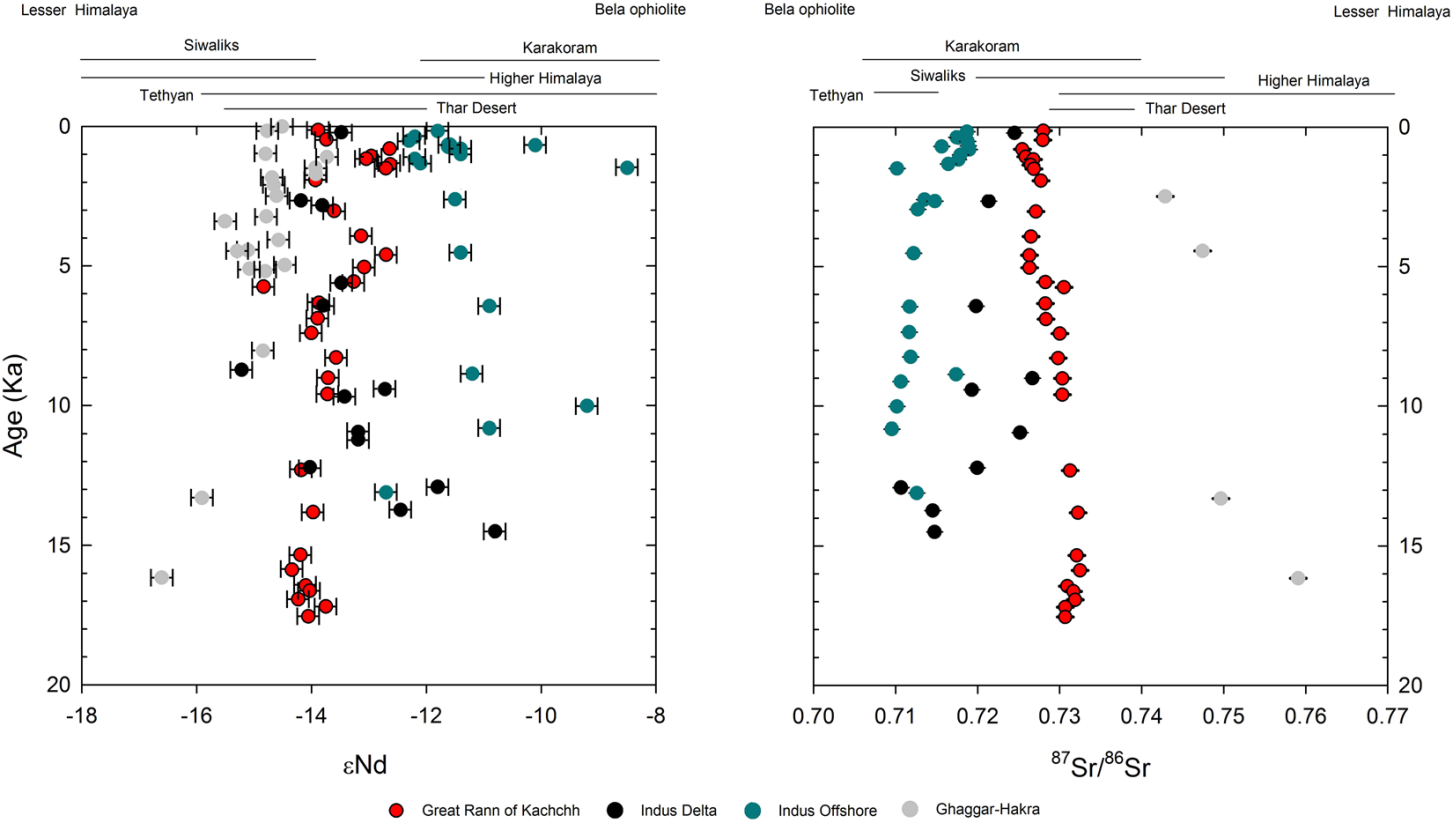
Deglacial and Holocene downcore variations in ε_Nd_ and ^87^Sr/^86^Sr ratio values for GRK sediments plotted along with Indus River, Indus Shelf and Ghaggar-Hakra system. Ranges of variability for potential sources are shown at the top of each graph. Graphs were prepared using a licensed copy of Sigma Plot v.10.

Marine sediments accumulating at the core location show a very tight range of variability within the Nd-Sr space (Fig. 2) The Aravalli sedimentary source is similar in isotopic composition to our sediments (present study; Fig. 2) but it cannot account for a significant contribution to the infilling of a large volume GRK basin. The mica-rich mineralogy of sediments (i.e., illite and chlorite) is indicative instead of their Himalayan and/or Karakoram origin^18, 28^. Similarly the Kachchh mainland hills were probably not a significant source of sediments given their small areal extent and geomorphology (i.e., the hydrographic network of short rivers is preferentially oriented southward). Therefore the remaining potential sediment sources for GRK sediments are Himalayan and/or Sub-Himalayan rivers including the Indus and the postulated Vedic Saraswati. In fact our sediments plot as a mixture of Indus sediments^18, 22^ with high ε_Nd_ and low radiogenic Sr typical for the Karakoram and low ε_Nd_ and high radiogenic Sr in Ghaggar-Hakra sediments^18, 22, 23^ indicative of High and Lesser Himalayan sources (Figs 2 and 3). Sediments coming from the Thar Desert^25, 33^ could also account for a significant contribution to GRK (Fig. 2), but this is not surprising as the Thar has been interpreted as a mixture of Himalayan and Sub-Himalayan sediments from the Indus and Ghaggar-Hakra systems^23^. However, Thar Desert is a vast sediment reservoir that is still poorly characterized geochemically.

### Sedimentation History

The temporal variability in Nd and Sr composition for the marine sediments at our GRK site is remarkably subdued (see Fig. 3) when compared to similar compositional histories of potential sources (i.e., Indus, Ghaggar-Hakra and Thar). However it is clear that GRK sediments before 10 ka plot between the two sources of sediments (Fig. 3) possibly indicating input from the Himalayas (Higher and/or Lesser) in addition to Indus sediments that include Karakoram and Tethyan Himalayan signals. A Thar Desert origin for the sediments is also possible but this vast region may exhibit a large variability in Nd and Sr isotopic composition that needs to be better assessed (Fig. 2). After that period the sediments in the GRK are practically indistinguishable from the Indus when using Nd and Sr fingerprinting. In contrast sediments from the Indus continental shelf, at least those located west of the Indus canyon that have been measured so far, appear more radiogenic in Nd and have lower ^87^Sr/^86^Sr values due to alongshore contributions from the Bela ophiolite^19, 22^. Thus another alternative interpretation explaining the divergence between GRK and Indus isotopic signatures before 10 ka could be that the Indus sediments themselves contain a significant contribution from the Bela Ophiolite before 10 ka (Fig. 3). Such an input could have come alongshore from the west into the GRK and Indus paleo-estuary when its delta was only incipiently developing more inland.

The rather invariant history of the GRK sediment composition since deglaciation contrasts with the Indus record (Fig. 3), which shows an increasing Lesser Himalayan input^22^. On the other hand Ghaggar-Hakra sediments show an increasingly Thar-like signature in the later Holocene (Fig. 3), a trend that cannot be recognized in the GRK sediments. In that case the GRK sediments could have been a mixture of Indus and Ghaggar-Hakra sediments since the beginning of our core records. The GRK record could also be interpreted to be strongly dominated by Thar sediments if we assume that a desert is somewhat homogenous (Fig. 3). As such, the signature for an independent Saraswati extending the course of the Ghaggar-Hakra towards the Arabian Sea cannot be discerned in the GRK using the Nd-Sr isotopic system. The most likely reason for that is not the mixing between Indus and Ghaggar-Hakra sediments *per se* but the input from the mixed Thar reservoir.

### Landscape Dynamics

Many courses for the Vedic Saraswati have been proposed over the years^4, 11, 34–36^ but they generally lack continuity in subsurface data and/or chronological information. Our new isotopic data suggests that a river, flowing parallel to and independent of the Indus, may have existed and reached the GRK before 10 ka (Fig. 3). At the time the Ghaggar-Hakra system may have been a much larger river tapping the Sutlej and/or the Yamuna^8, 10, 33, 37, 38^. However, this interpretation is dependent on the isotopic homogeneity of the vast sediment reservoir of the Thar Desert, which is still to be assessed. Whether such a river reached the Arabian Sea via the GRK during the Holocene and especially, during Vedic times remains to be demonstrated.

Recent studies of the upper courses of the proposed Saraswati in Haryana and Cholistan suggested that river desiccation started ~6.5 ka B.P.^8, 10, 24, 34, 38^. However, Giosan *et al*.^8^ showed that fluvial sedimentation was still active in the western part of the Thar Desert as late as ~3 ka, with river courses joining the Nara valley. Currently we do not know if the Nara was independent or received input from the Indus near Sukkur or further down after emerging from below the Rohri Hills (Fig. 1). However, the isotopic composition of the GRK sediments are not in contradiction with the idea of a dwindling Ghaggar-Hakra-Nara under the aridification of South Asia as the monsoon declined in the late Holocene^39–41^.

The Holocene sedimentation pattern in the GRK basin shows a regressive pattern with the basin becoming shallower as the sea level rose and rivers provided infill. During the deglaciation when sea level was considerably lower, a Saraswati-like river had a better chance to deliver a pure signal to GRK if it possessed its own Pleistocene incised valley, independent of the Indus incised valley^10, 22, 41^. However, by ~5 to 6 ka the Indus delta extended into the western GRK and probably provided sediments directly into the GRK^41^. Historical maps and documents^42–45^ suggest that GRK may have still been a gulf ca. 500 years ago^5, 46^. A deeper GRK with fresh water input from the Indus and potentially Ghaggar-Hakra-Nara would have provided a more productive marine environment and navigable ways for the old coastal Harappan towns in the region (e.g, Dholavira) as well as for later historical settlements.

## Conclusions

The Nd and Sr isotopic composition of sediments from our Dhordo core site in the Great Rann of Kachchh suggests that a large Himalayan or Sub-Himalayan Saraswati-like river may have discharged into the Arabian Sea until 10 ka. However, our study also shows that radiogenic isotope fingerprinting of the GRK sediments is unlikely to detect a gradually drying Saraswati-like river after that time, due to contamination with sediments from the Thar Desert and/or the Indus. Alternatively the Thar may have been the dominant sediment source along with the Indus for the entire post-glacial history of the GRK. Future studies should concentrate instead on geophysical imaging, dating and geochemical fingerprinting of subsurface deposits from infilled channels along potential river courses in the Thar Desert. However, the Holocene sedimentary evolution of the Great Rann should be better explored to understand its role in Harappan and historical coastal habitation.

### Sampling and methodology

The continuous sediment core was raised from the GRK basin (Fig. 1; SI Fig. 1). A ~60 m long core was drilled from the central part of the basin at Dhordo (23°49′37.9” N and 69°36′09.9” E). The entire core section was then X-radiographed before it was opened. The core pipes were then split longitudinally into two halves: one half of the core was sampled at 2 cm intervals while the other half was preserved as an archive. The GRK sediments are typically fine-grained, dominated by silts and clays with occasional sands^6^ (SI Fig. 1). The samples obtained from our cores at various depth intervals were analyzed for Nd-Sr radiogenic isotopes (SI Table 1) and radiocarbon chronologies (reported in Khonde *et al*.^28^). We also collected samples for Sr-Nd measurements from the Luni and Rupen rivers and the local stream Saraswati that discharge into the Great and Little Rann basins from the east. These rivers come from the Aravalli Hills, which lie further to the east and northeast.

### Nd and Sr isotopic systematics

Measurements were carried out on carbonate- and organic matter-free silicate fraction. A known amount (~100 mg) of this fraction taken in Teflon vials (Savillex) was spiked with ^84^Sr and ^150^Nd and subjected to acid digestion with concentrated HF-HNO_3_-HCl at 90 °C to complete dissolution. Pure Nd and Sr fractions were separated from the solution following standard ion exchange procedures^17, 47^. The fractions were then dried and redissolved in 4 ml of 0.4 N HNO_3_. Both Nd and Sr measurements were done on MC-ICP-MS in static multi-collection mode at PRL^15, 17^. The measured ^87^Sr/^86^Sr and ^143^Nd/^144^Nd ratios were corrected for instrumental mass fractionation by normalizing them with ^86^Sr/^88^Sr, 0.1194 and ^146^Nd/^144^Nd, 0.7219. The Nd and Sr concentrations for these samples were obtained by isotope dilution method. A standard solution of 200 ppb of SRM 987 Sr-standard was measured several times on MC-ICP-MS that yielded an average value of 0.710307 ± 0.000010 (1σ, n = 10) for ^87^Sr/^86^Sr whereas ^143^Nd/^144^Nd in 100 ppb solution of JMC standard yielded 0.511732 ± 0.000016 (1σ, n = 10) respectively. Replicate samples were also measured for Nd and Sr concentrations and isotopic compositions on selected samples to check the overall reproducibility of the Nd-Sr measurements (SI Tables 2 and 3). Based on replicate measurements, the average variation was found to be 0.0002 and 0.2 for ^87^Sr/^86^Sr and ε_Nd_ respectively. However the standard errors for ^87^Sr/^86^Sr and^143^Nd/^144^Nd are 0.0014%, 0.0010% respectively.

## Electronic supplementary material

Supplementary Datasheet 1
