# Supplementary material for: Tracing the Vedic Saraswati River in the Great Rann of Kachchh
Source: Sci Rep. 2017 Jul 14;7:5476. doi: 10.1038/s41598-017-05745-8 (PMC5511136; doi:10.1038/s41598-017-05745-8)
Supplement: Supplementary file 1 — Supplementary Datasheet 1 [file 41598_2017_5745_MOESM1_ESM.doc]

**Supplementary information for**

**Tracing the Vedic Saraswati River**

**in the Great Rann of Kachchh**

Nitesh Khonde^1, #^, Sunil Kumar Singh^2^, D. M. Maurya^1*^,

Vinai K. Rai^2^, L. S. Chamyal^1^ and Liviu Giosan^3^

^1^Department of Geology, The M. S. University of Baroda, Vadodara 390002, India.

^2^Physical Research Laboratory, Navrangpura, Ahmedabad 380009, India.

^3^Woods Hole Oceanographic Institution, Woods Hole, MA 02543, USA

^#^Present Address: Birbal Sahni Institute of Palaeosciences, Lucknow 266007, India

Corresponding Author: dmmaurya@yahoo.com

**Supplementary Figures:**

**SI Figure 1.** a. Lithology and grain size measurements for the Dhordo core (after Maurya et al. 2013). AMS ^14^C-dated horizons are indicated by arrows (after Khonde et al. 2016). Graphs were prepared using licensed copy of Coral Draw v.15.

**
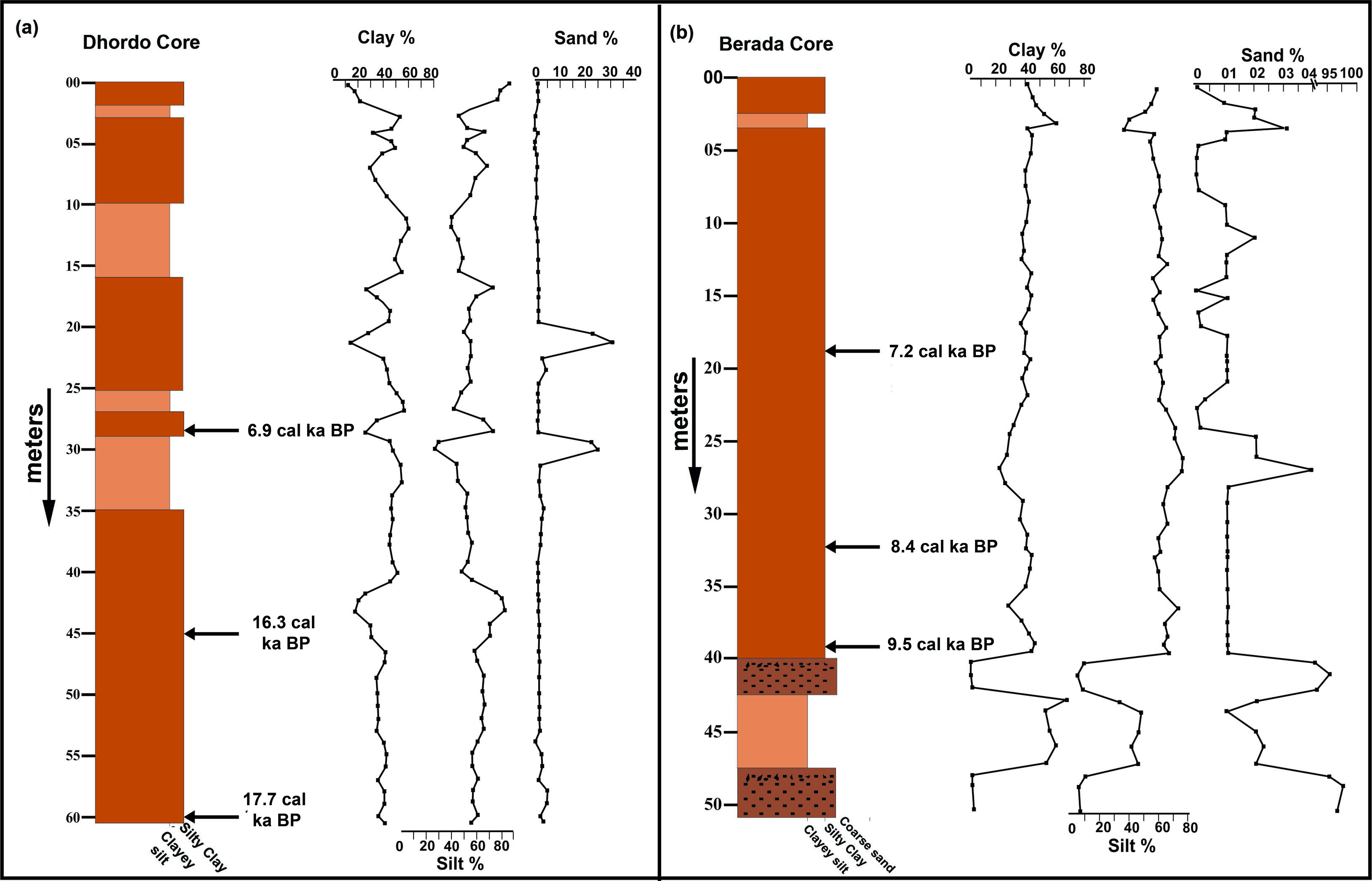
**

**Table 1. Downcore variations in the ^87^Sr/^86^Sr, Sr (ppm), ^143^Nd/^144^Nd, ε_Nd_ and Nd (ppm) isotopic concentration in the Dhordo (DH) Core in the Great Rann basin of Kachchh, and rivers draining from the Aravallis.**

| **Sr No** | **Sample ID** | **Depth (m)** | **^87^Sr/^86^Sr (Corr.)** | **Sr (ppm)** | **^144^Nd/^143^Nd** | **Nd (ppm)** | **ε_Nd_**  **_(CHUR0)_** |
| --- | --- | --- | --- | --- | --- | --- | --- |
| **01** | DH-1 | 0.48 | 0.728006 | 140.35 | 0.511926 | 17.33 | -13.88 |
| **02** | DH-2 | 1.72 | 0.727946 | 121.22 | 0.511934 | 16.95 | -13.74 |
| **03** | DH-3 | 2.94 | 0.725449 | 99.33 | 0.511990 | 14.87 | -12.64 |
| **04** | DH-4 | 3.93 | 0.725854 | 97.41 | 0.511974 | 14.73 | -12.96 |
| **05** | DH-5 | 4.29 | 0.726797 | 105.47 | 0.511969 | 15.45 | -13.04 |
| **06** | DH-6 | 5.02 | 0.726484 | 100.23 | 0.511991 | 15.02 | -12.63 |
| **07** | DH-7 | 5.54 | 0.726849 | 100.26 | 0.511987 | 14.75 | -12.70 |
| **08** | DH-8 | 7.1 | 0.727716 | 120.27 | 0.511924 | 16.39 | -13.93 |
| **09** | DH-9 | 11.18 | 0.727097 | 117.72 | 0.511941 | 17.16 | -13.60 |
| **10** | DH-10 | 14.48 | 0.726502 | 111.47 | 0.511965 | 14.95 | -13.14 |
| **11** | DH-11 | 16.96 | 0.726320 | 112.25 | 0.511987 | 15.35 | -12.70 |
| **12** | DH-12 | 18.61 | 0.726340 | 101.91 | 0.511967 | 15.14 | -13.08 |
| **13** | DH-13 | 20.52 | 0.728226 | 117.25 | 0.511958 | 16.39 | -13.26 |
| **14** | DH-14 | 21.2 | 0.730531 | 148.40 | 0.511877 | 17.20 | -14.84 |
| **15** | DH-15 | 23.34 | 0.728269 | 101.74 | 0.511927 | 15.53 | -13.87 |
| **16** | DH-16 | 25.38 | 0.728236 | 98.40 | 0.511926 | 15.07 | -13.89 |
| **17** | DH-17 | 26.64 | 0.730023 | 107.46 | 0.511920 | 16.57 | -14.00 |
| **18** | DH-18 | 28.44 | 0.729822 | 106.99 | 0.511943 | 16.18 | -13.57 |
| **19** | DH-19 | 29.93 | 0.730340 | 108.24 | 0.511935 | 15.25 | -13.71 |
| **20** | DH-20 | 31.13 | 0.730340 | 108.24 | 0.511935 | 17.23 | -13.72 |
| **21** | DH-21 | 36.67 | 0.731281 | 112.06 | 0.511911 | 18.99 | -14.18 |
| **22** | DH-22 | 39.77 | 0.732244 | 106.06 | 0.511922 | 16.86 | -13.97 |
| **23** | DH-23 | 42.91 | 0.732095 | 125.83 | 0.511911 | 15.43 | -14.19 |
| **24** | DH-24 | 44.01 | 0.732493 | 109.12 | 0.511903 | 16.00 | -14.34 |
| **25** | DH-25 | 46.15 | 0.730941 | 109.83 | 0.511915 | 17.22 | -14.10 |
| **26** | DH-26 | 48.22 | 0.731680 | 109.28 | 0.511919 | 16.63 | -14.03 |
| **27** | DH-27 | 51.57 | 0.731885 | 107.09 | 0.511908 | 17.33 | -14.23 |
| **28** | DH-28 | 54.5 | 0.730690 | 114.45 | 0.511933 | 17.21 | -13.75 |
| **29** | DH-29 | 58.45 | 0.730690 | 114.45 | 0.511918 | 17.41 | -14.05 |
| **30** | LUNI | Aravalli river | 0.730103 | 131.19 | 0.511922 | 4.48 | -13.97 |
| **31** | RUPEN | Aravalli river | 0.731141 | 83.97 | 0.511876 | 17.71 | -14.86 |
| **32** | SARASW-ATI | Aravalli river | 0.734564 | 57.80 | 0.511858 | 5.60 | -15.22 |

**Table 2. Reproducibility of elemental and isotopic composition of Sr in the sediments analysed**.

| Sample ID | ^87^Sr/^86^Sr | ^87^Sr/^86^Sr  Std Error % | Sr (ìg/g) | Sr (ìg/g)  Std Error % |
| --- | --- | --- | --- | --- |
| DH-5-48 | 0.72680 | 0.0016 | 121.2 | 0.0050 |
|  | 0.72671 | 0.0011 | 120.9 | 0.0061 |
| DH-7-62 | 0.72690 | 0.0021 | 117.5 | 0.0084 |
|  | 0.72710 | 0.0009 | 117.7 | 0.0042 |
| DH-11-49 | 0.73053 | 0.0011 | 148.4 | 0.0060 |
|  | 0.73082 | 0.0012 | 147.9 | 0.0033 |
| DH-14-29 | 0.72959 | 0.0033 | 107.2 | 0.0142 |
|  | 0.72974 | 0.0025 | 107.3 | 0.0090 |
| LUNI | 0.72992 | 0.0009 | 131.5 | 0.0029 |
|  | 0.73010 | 0.0022 | 131.2 | 0.0066 |

**Table 3. Reproducibility of elemental and isotopic composition of Nd in sediments analysed.**

| Sample ID | ^143^Nd/^144^Nd | ^143^Nd/^144^Nd Std Error % | ºNd | ºNd  Std Error % | Nd (ìg/g) | Nd (ìg/g)  Std Error % |
| --- | --- | --- | --- | --- | --- | --- |
| DH-5-48 | 0.51199 | 0.0019 | -12.7 | 1.5 | 14.7 | 0.0044 |
|  | 0.51198 | 0.0008 | -12.9 | 0.6 | 14.8 | 0.0028 |
| DH-11-49 | 0.51188 | 0.0007 | -14.8 | 0.5 | 17.2 | 0.0018 |
|  | 0.51190 | 0.0008 | -14.5 | 0.5 | 14.6 | 0.0020 |
| DH-14-29 | 0.51196 | 0.0012 | -13.3 | 0.9 | 16.4 | 0.0027 |
|  | 0.51193 | 0.0011 | -13.7 | 0.8 | 16.4 | 0.0036 |
| RUPEN | 0.51188 | 0.0005 | -14.8 | 0.3 | 17.7 | 0.0015 |
|  | 0.51188 | 0.0005 | -14.9 | 0.3 | 17.7 | 0.0015 |
